# Supplementary material for: A protein phosphatase network controls the temporal and spatial dynamics of differentiation commitment in human epidermis
Source: eLife. 2017 Oct 18;6:e27356. doi: 10.7554/eLife.27356 (PMC5667932; doi:10.7554/eLife.27356)

Mishra et al.
Supplementary File 14

P-Erk1/2 (42, 44 kDa)

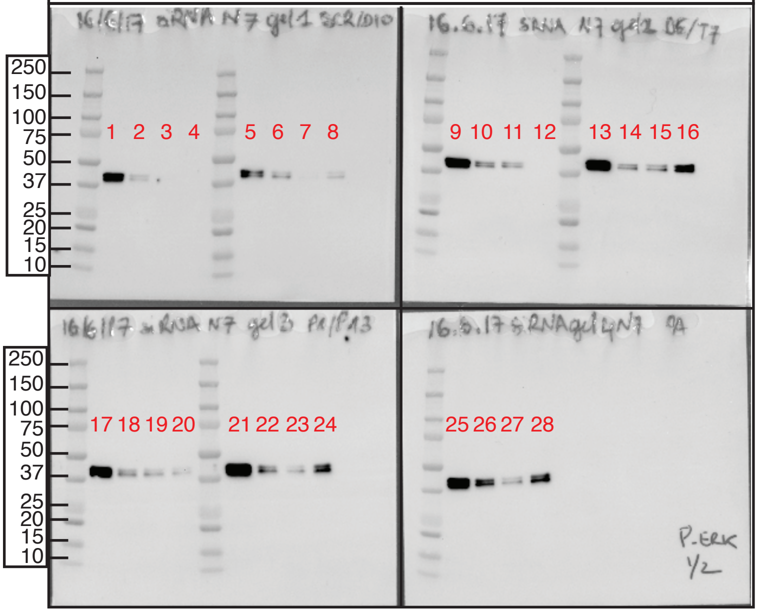

Erk1/2 (42, 44 kDa) + Cyclophilin B (20 kDa)

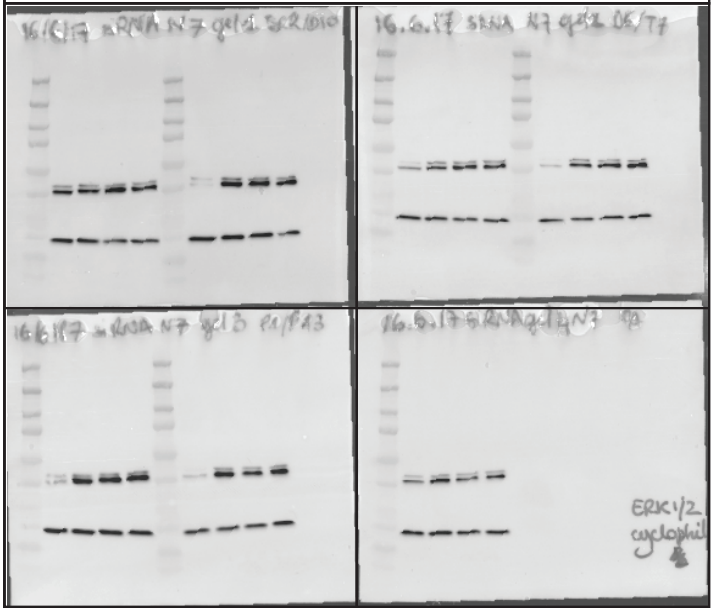

P-p38 (43 kDa)

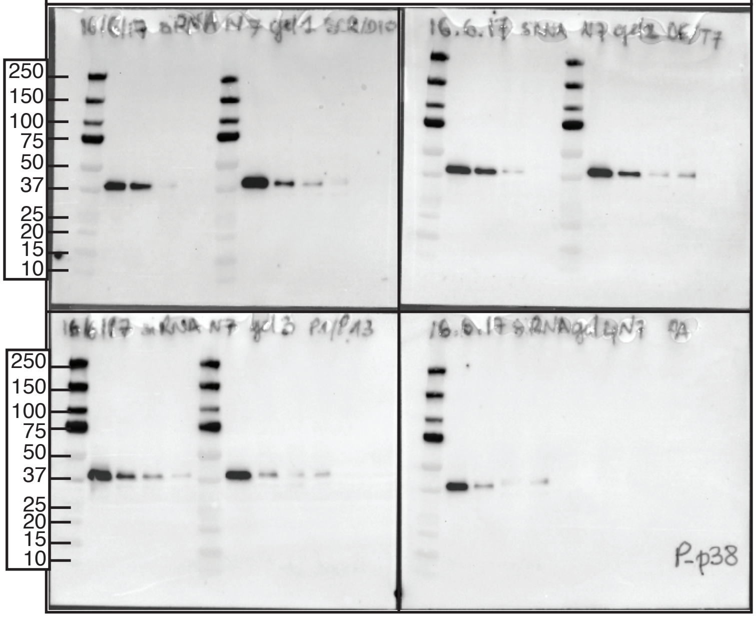

p38 (43 kDa)

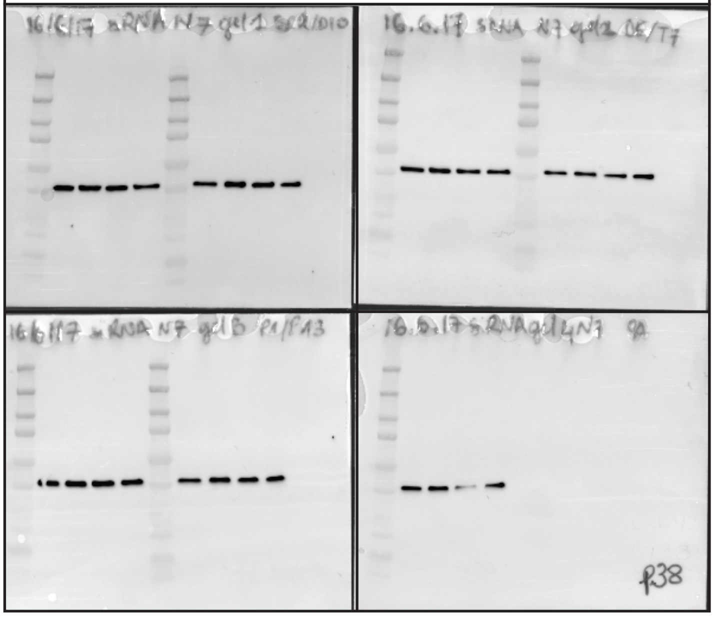

1: siSCR 0h	5: siDUSP10 0h	9: siDUSP6 0h	13: siPPTC7 0h	17: siPTPN1 0h	21: siPTPN13 0h	25: siPPP3CA 0h
2: siSCR 4h	6: siDUSP10 4h	10: siDUSP6 4h	14: siPPTC7 4h	18: siPTPN1 4h	22: siPTPN13 4h	26: siPPP3CA 4h
3: siSCR 8h	7: siDUSP10 8h	11: siDUSP6 8h	15: siPPTC7 8h	19: siPTPN1 8h	23: siPTPN13 8h	27: siPPP3CA 8h
4: siSCR 12h	8: siDUSP10 12h	12: siDUSP6 12h	16: siPPTC7 12h	20: siPTPN1 12h	24: siPTPN13 12h	28: siPPP3CA 12h

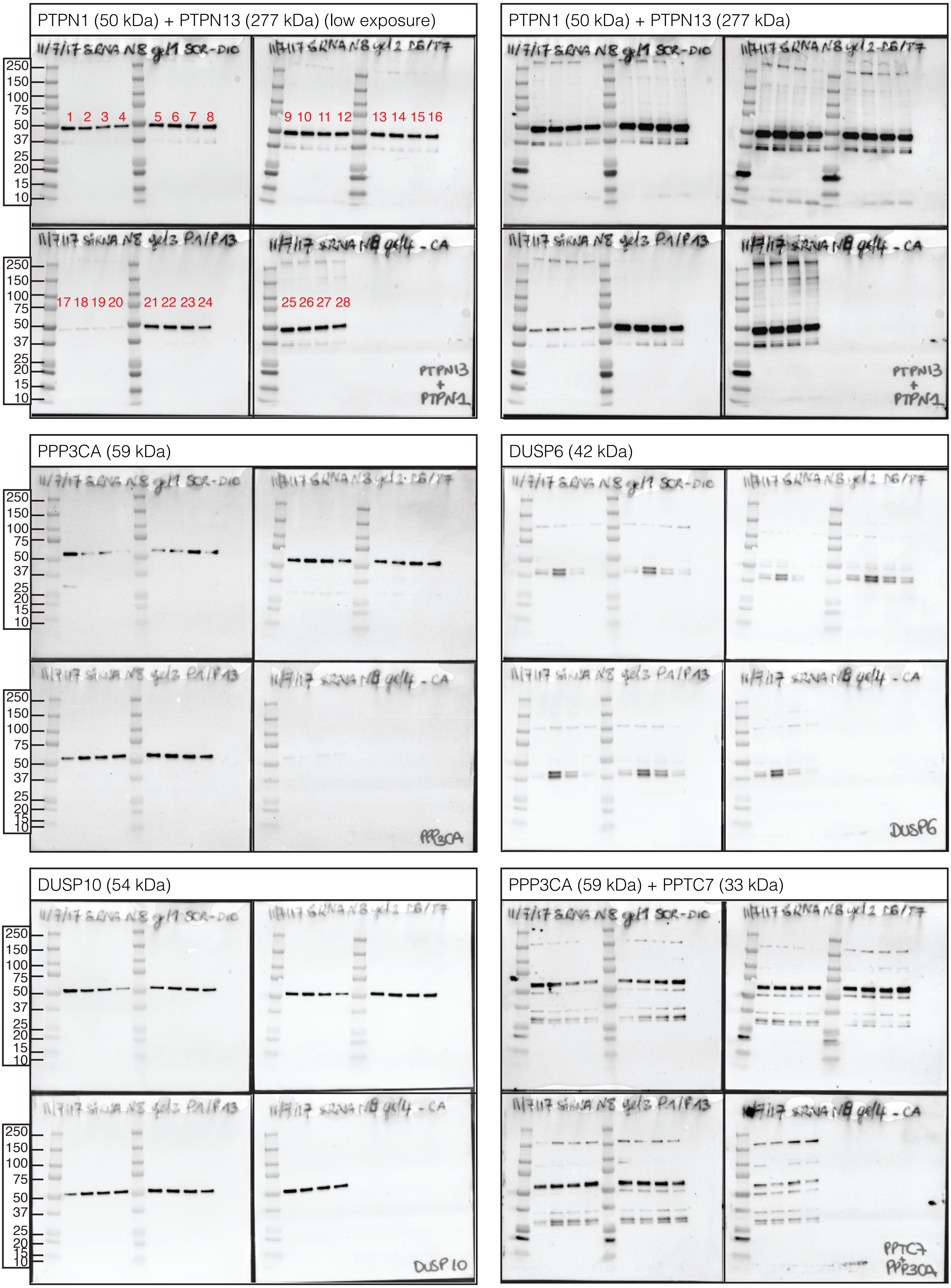

1: siSCR 0h	5: siDUSP10 0h	9: siDUSP6 0h	13: siPPTC7 0h	17: siPTPN1 0h	21: siPTPN13 0h	25: siPPP3CA 0h
2: siSCR 4h	6: siDUSP10 4h	10: siDUSP6 4h	14: siPPTC7 4h	18: siPTPN1 4h	22: siPTPN13 4h	26: siPPP3CA 4h
3: siSCR 8h	7: siDUSP10 8h	11: siDUSP6 8h	15: siPPTC7 8h	19: siPTPN1 8h	23: siPTPN13 8h	27: siPPP3CA 8h
4: siSCR 12h	8: siDUSP10 12h	12: siDUSP6 12h	16: siPPTC7 12h	20: siPTPN1 12h	24: siPTPN13 12h	28: siPPP3CA 12h

Cyclophilin B (20 kDa)

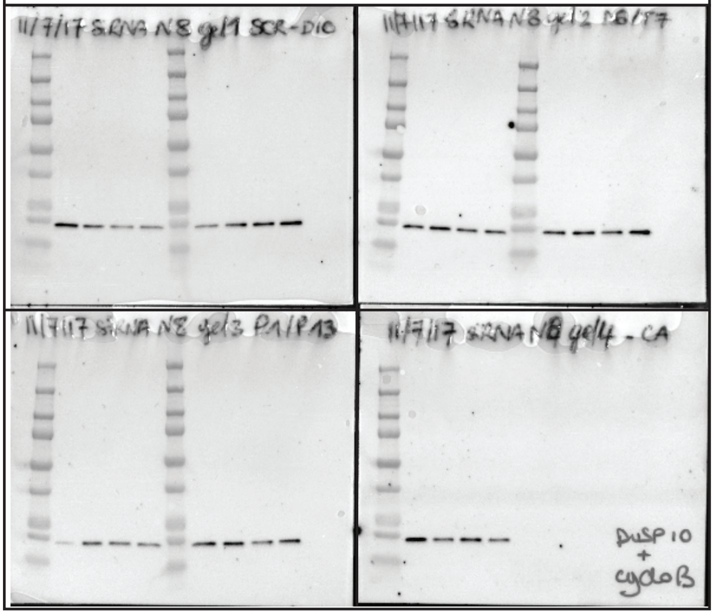

Supplement: Supplementary file 14. [file elife-27356-supp14.pdf]
